# Wilkie's Syndrome: An Unexpected Finding

**DOI:** 10.7759/cureus.20413

**Published:** 2021-12-14

**Authors:** Mariana Claro, Diogo Sousa, Alberto Abreu da Silva, João Grilo, José Augusto Martins

**Affiliations:** 1 General Surgery, Unidade Local de Saúde do Litoral Alentejano, Santiago do Cacém, PRT

**Keywords:** wilkie's syndrome, post-prandial abdominal pain, duodenojejunostomy, intestinal obstruction, superior mesenteric artery syndrome

## Abstract

Wilkie's syndrome is a rare pathology caused by extrinsic compression of the third part of the duodenum by the superior mesenteric artery (SMA) at its origin. The symptoms are variable and non-specific consisting of postprandial abdominal pain, nausea and vomiting, early satiety, anorexia, and weight loss. A contrast-enhanced CT scan is the standard imaging modality. Surgery is reserved for severe cases or those unresponsive to pharmacological treatment.

We present a case of a 66-year-old woman with a history of prolonged postprandial abdominal pain, nausea, and substantial weight loss (30 kg in four months). Laboratory tests revealed acute renal failure with hypokalemia due to severe dehydration. She underwent an abdominal-pelvic CT scan that showed enlargement of the second and third parts of the duodenum, without an identifiable cause, followed by an upper gastrointestinal (GI) endoscopy that showed a dilated duodenum until D2 and inability of progression to D3, without mucosal abnormalities. Her MRI revealed considerable gastric and proximal duodenum distension with compression of D3 by the SMA. The patient underwent a laparoscopic duodenojejunostomy with intra-operative findings consistent with the diagnosis. The procedure and the postoperative period were uneventful, and the patient was discharged on the ninth postoperative day. Gastrografin study made at day six postop showed normal progression of the oral contrast. At the outpatient reevaluation one month postop, she remained asymptomatic and with progressive weight gain.

Wilkie's syndrome is a rare form of intestinal obstruction, which is commonly disregarded. Its non-specific symptoms make it a challenging diagnosis and imply a high clinical suspicion. Among the different surgical options, duodenojejunostomy presents the best outcomes.

## Introduction

Compression of the anterior wall of the duodenum's third part by the aortomesenteric angle narrowing as a cause of gastrointestinal obstruction was first described by Carl Von Rokitansky in 1861 in a postmortem case, which was later studied in detail by Wilkie in 1927 [1].

This syndrome, known as Wilkie's syndrome, superior mesenteric artery syndrome (SMA syndrome), or cast syndrome, is very rare with only 500 reported cases in the literature and an estimated prevalence of 0.013%-0.3%. It is more common among the female gender (2:1 ratio) [2] between the second and fourth decades of life [3].

Several causes may be responsible for this syndrome, ranging from congenital anatomical abnormalities to hypercatabolic or malnutrition states. Independently of the cause, a loss of the perivascular and retroperitoneal fatty cushion occurs, causing a narrowing of the aortomesenteric angle and subsequently external duodenal compression [3]. Nevertheless, 40.4% of cases are idiopathic [4].

The duodenal compression can be partial or complete, acute or chronic, resulting in symptoms varying from vague postprandial epigastric pain and nausea to recurrent postprandial vomiting with severe weight loss and electrolyte imbalances. Conservative measures such as positional postprandial body changes, fluid and electrolyte correction, and hypercaloric nutritional therapy [5] should be attempted first with surgery being indicated for more severe or clinically persistent cases.

This article was previously presented as a meeting poster at XLI National General Surgery Congress on 17th and 18th June 2021, Figueira da Foz, Portugal.

## Case presentation

The authors present a case of a 66-year-old woman who presented to the emergency department (ER) with a history of prolonged postprandial abdominal pain, nausea, vomiting, and substantial weight loss (30 kg in four months) with a BMI of 14.7 kg/m^2^. Her past medical history included hypertension, dyslipidemia, and past infection with severe acute respiratory syndrome coronavirus 2 (SARS-CoV-2) that was deemed cured in January 2021. She had previously been submitted to a hysterectomy for uterine myomatosis.

Her physical examination revealed moderate dehydration with no other positive findings. Her laboratory tests were consistent with prerenal acute kidney injury as well as mild hypokalemia and hyponatremia due to persistent vomiting. Her albumin and total protein levels were within the normal range (Table 1).

**Table 1:** Blood test results

Blood Test Analysis	
White blood cell count	8.1 x 103/µL (4.0-11.00)
Neutrophils (%)	74.1% (40.0-75.0)
Hemoglobin	13.6 g/dl (11.7-15.5)
Platelets	312 000 (150-400)
Urea	165 mg/dl (<43)
Creatinine	2.6 mg/dl (0.7-1.1)
Sodium	130 mEq/L (136-146)
Potassium	3.4 mEq/L (3.5-5.1)
Total protein	7.6 g/dl (6.6-8.3)
Albumin	4.5 g/dl (3.5-5.2)
C-reactive protein (CRP)	4.06 mg/dl (<0.50)

**Figure 1:**
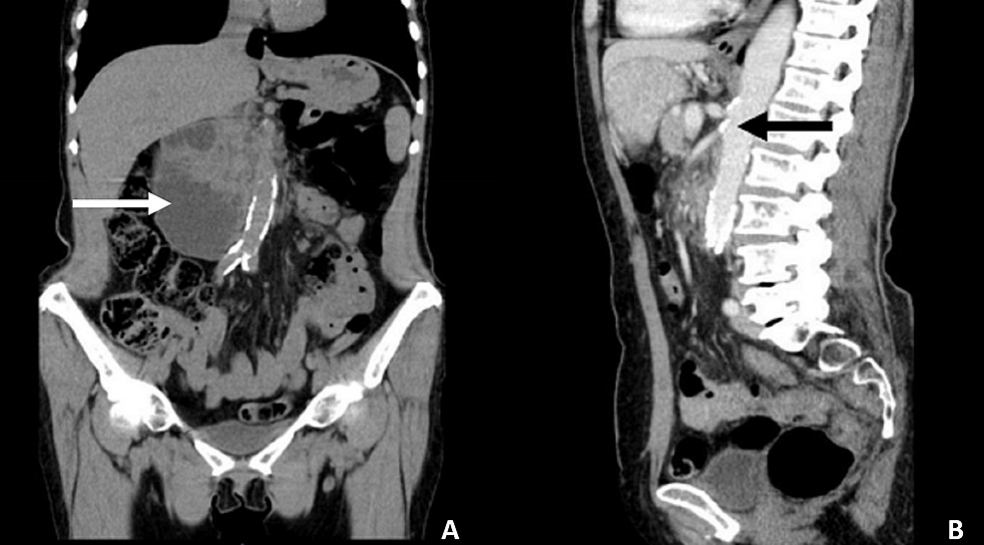
Contrast-enhanced abdominal-pelvic CT scan (A) Coronal view: white arrow evidencing duodenal arch dilation. (B) Sagittal view: black arrow signaling the aortomesenteric angle.

She was admitted for electrolyte disorders' correction and continuation of the etiological study. Abdominal-pelvic CT scan with IV contrast showed dilation of the second and third parts of the duodenum, without an identifiable cause (Figure 1).

**Figure 2:**
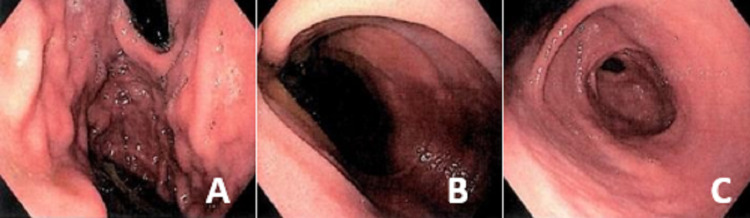
Upper GI endoscopy (A) Normal gastric morphology. (B) Dilation of the duodenal bulb and D2 without mucosal abnormalities. (C) Failure of endoscopic progression to D3.

She was then submitted to an upper GI endoscopy that showed a dilated duodenum until D2 and failure of endoscopic progression to D3, without identifiable mucosal abnormalities (Figure 2).

**Figure 3:**
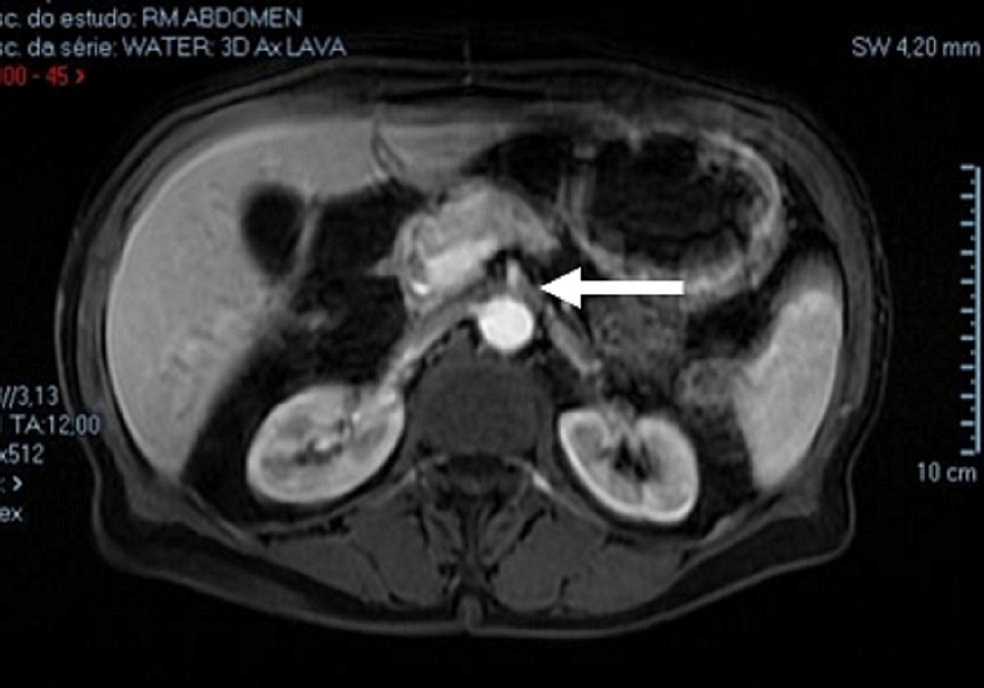
Abdominal MRI Considerable gastric and proximal duodenum distension until the aortomesenteric angle with collapsed distal duodenum. White arrow signalizing the aortomesenteric angle.

Finally, an abdominal pelvic MRI revealed considerable gastric and proximal duodenum distension until the aortomesenteric angle with collapsed distal duodenum suggestive of superior mesenteric artery syndrome (Figure 3).

**Figure 4:**
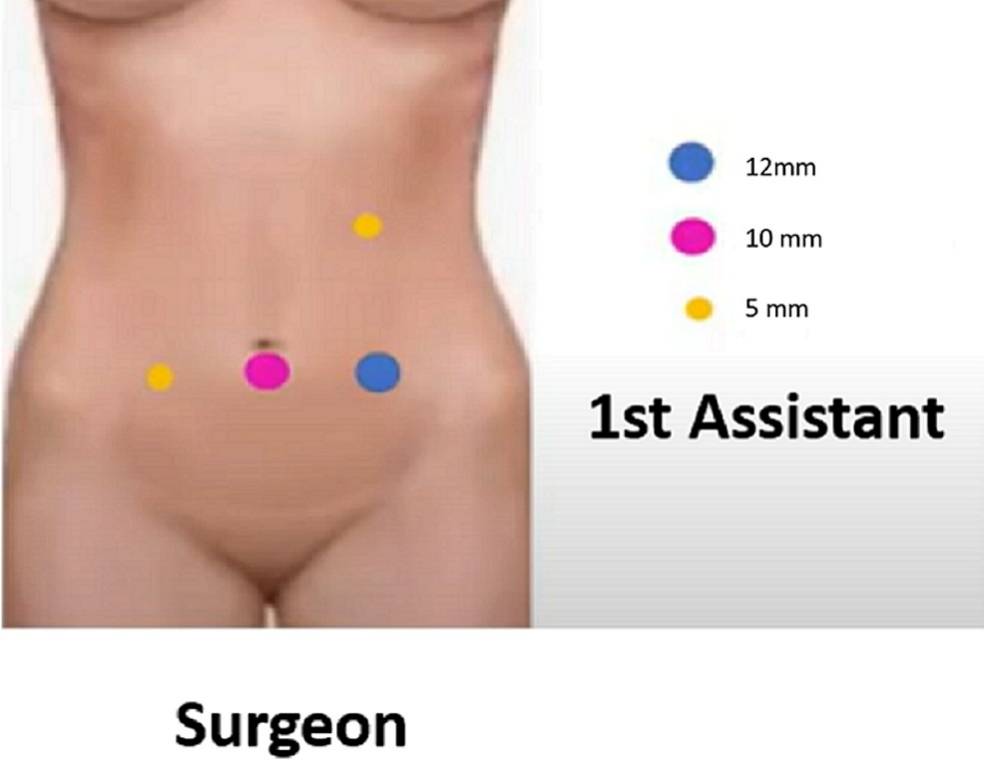
Port placement Image obtained and modified from MESDA Channel: Laparoscopic Duodenojejunostomy for SMA syndrome by Dr. Kamthorn Yolsuriyanwong, PSU, Thailand [6]. Permission was obtained from the original publisher.

Since her symptomatology was intense and prolonged characterized by severe weight loss and food intolerance that persisted despite dietary modification and use of prokinetic agents, surgery was contemplated. A laparoscopic duodenojejunostomy was then proposed to the patient to which she consented. She was placed in a supine position with the legs abducted. The surgeon stood between the patient's legs, and the first assistant stood at the patient's left side. Four trocars were placed: two 5-mm trocars on the left upper quadrant and the right flank, and a 12-mm port was placed on the left flank. A 30º scope was used on the umbilical port (Figure 4).

**Figure 5:**
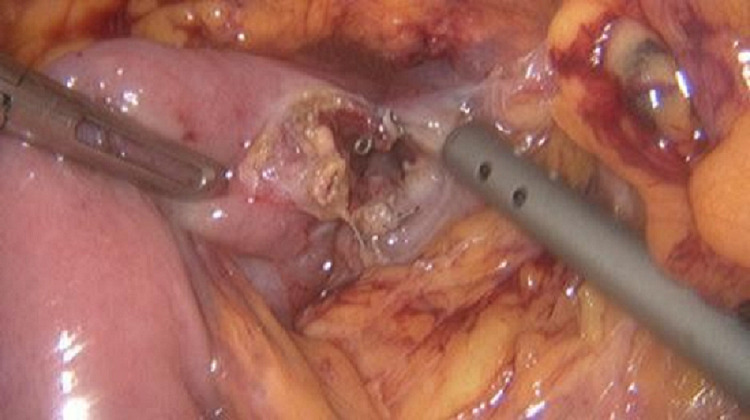
Laparoscopic duodenojejunostomy Mechanical side-to-side duodenojejunostomy with a 3.5-mm endoGIA. The common enterotomy was closed with an absorbable monofilament running suture. EndoGIA, Endovascular gastrointestinal anastomosis.

The inspection of the abdominal cavity proved there was a distended duodenum up until the origin of the superior mesenteric artery. The retroperitoneum was opened, and the ligament of Treitz was identified. A portion of the proximal jejunum, approximately 20 cm distal to the ligament of Treitz, was found to easily reach the duodenum. A duodenotomy and jejunotomy were made to perform a mechanical side-to-side anisoperistaltic duodenojejunostomy with a 3.5-mm endoGIA (Figure 5). The common enterotomy was closed with an absorbable monofilament running suture. A passive 10-mm silicone drain was placed near the anastomosis. The trocar sites were closed in the standard fashion.

**Figure 6:**
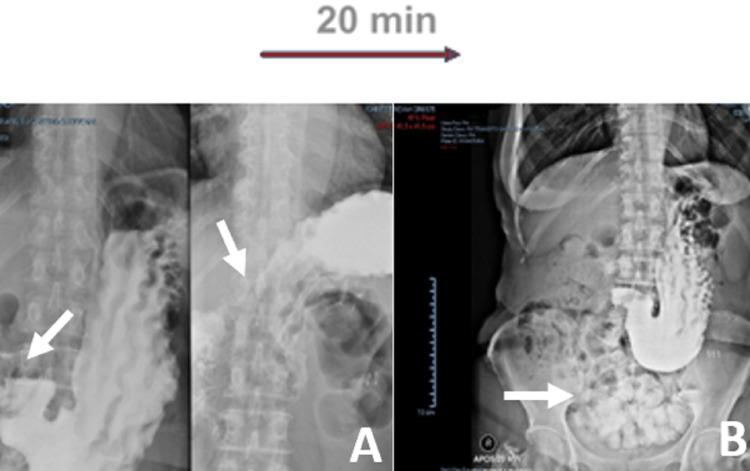
Upper GI study with Gastrografin Progression of the oral contrast to the jejunum within 20 minutes. (A) The difficulty of contrast progression from D2 to D3. (B) Contrast within jejunal and duodenal loops made through the duodenojejunostomy. Images were taken 20 minutes apart.

The procedure and the postoperative period were uneventful. Gastrografin study at day six postop had normal progression of the oral contrast without signs of stasis or obstruction (Figure 6).

She was discharged on the ninth postoperative day completely asymptomatic and tolerating oral intake. At the outpatient re-evaluation after one-month postop, she remained asymptomatic and with progressive weight gain.

## Discussion

Wilkie's syndrome is characterized by a narrowing of the aortomesenteric angle. In a physiologic state and due to the erect position, the aorta-SMA angle ranges from 38º to 65º [7] and is maintained by the presence of perivascular fat tissue. As for the normal aortomesenteric distance, it lies between 10 and 28 mm [7]. In SMA syndrome, the angle acutely decreases ranging from 6º to 16º as well as the distance that can shorten until 2 mm, leading to extraluminal compression of the duodenum [7].

Etiological factors can be either congenital or acquired. Congenital causes include abnormally short or high insertion of the ligament of Treitz, dislocating the duodenum to a cranial position; a low insertion of the SMA, or peritoneal adhesions [8]. Acquired factors can be multifactorial. Catabolic states such as tumors or burns and diseases causing severe weight loss such as anorexia nervosa or malabsorption syndrome have been referred to as potential causes. Severe trauma or lesions associated with prolonged bedtime rest like brain trauma or spinal cord injuries have also been implied. Finally, postoperative states such as spinal surgery (cast syndrome), esophagectomies, or abdominal aorta aneurism repairs can also be in the genesis of this syndrome [8].

Retroperitoneal inflammatory thickening after acute pancreatitis has also been known to be a contributing factor to Wilkie's syndrome. Regardless of the associated factors, a depletion of the fatty cushion around SMA occurs, leading to the anatomical alterations mentioned above. In our case, the patient had a prolonged history without the identification of a specific trigger. She had no history of psychiatric disorders and she did not have any major past surgeries or conditions that forced her to be on lengthy bedtime rest. Hence we assumed that, as Proaño et al. stated [4], her etiology was idiopathic.

Patients with SMA syndrome may present acutely, with chronic insidious symptomatology or with an acute exacerbation of chronic symptoms [9]. Acute presentation is usually less common and is characterized by signs and symptoms of intestinal obstruction, being more prone to complications such as severe aspiration pneumonia. Chronic cases, like the one we presented here, can present with insidious, progressive, nonspecific symptoms that can last for years or decades. Epigastric intermittent postprandial pain (59%), nausea (50%), biliary or food content vomits (sometimes in a projectile manner), vague postprandial indisposition, early satiety (32%), gastric distension, gastroesophageal reflux, regurgitation, food intolerance, and weight loss [8] are the most commonly referred. Persistent vomiting can also lead to dehydration, severe hypovolemia, oliguria, electrolyte imbalances such as hypokalemia and metabolic alkalosis, and eventually Mallory Weiss lesions [8]. Symptoms can aggravate with meals and prone position and alleviate with lateral left decubitus or genupectoral position [3].

These symptoms can also mimic other conditions like pancreatitis, peptic ulcers, retroperitoneal or duodenal tumors, eating disorders, or disorders characterized by slow peristalsis such as dermatomyositis or systemic lupus erythematosus [4,8]. On physical examination, findings are usually vague but can include a distended abdomen, tender epigastrium on deep palpation, and high-pitched bowel sounds [7], none of which were exhibited by our patient.

Due to the low specificity of the signs and symptoms, clinical diagnosis requires a high index of suspicion especially in a patient who presents with postprandial abdominal pain, vomiting, and a recent history of significant weight loss [10]. Hence, diagnosis is made based not only on clinical evidence but also on radiological findings.

The diagnostic investigation often starts with a plain abdominal x-ray that, despite its low sensitivity, can reveal a proximal duodenal dilation and absence of distal bowel gas [2,7]. The upper GI endoscopy is usually performed to exclude an intrinsic mechanical bowel obstruction. In cases of SMA syndrome, the only positive finding given by the upper GI endoscopy is an extrinsic regular and sometimes pulsatile compression of the duodenal wall in the transition of D2 to D3. Barium radiography shows pathognomonic features such as dilation of the first and second parts of the duodenum with or without gastric dilation; obstruction of the third part of the duodenum and a clear cut line that demarks the obliteration of the duodenal lumen by the SMA; antiperistaltic flow of the barium proximal to the obstruction; and a delay of four to six hours in gastroduodenal transit time, with relief of the obstruction when the patient is placed in the knee-chest position, left lateral decubitus, or with the Hayes maneuver [1,7-9]. However, contrast-enhanced abdominal CT scan and MRI have emerged as the gold standard modalities for diagnosis [7] since they are non-invasive and provide crucial information like the aortomesenteric angle and distance, the extent of duodenal distension and exact point of obstruction, the assessment of the amount of retroperitoneal fat, and the exclusion of other frequent causes for intestinal obstruction (tumors, annular pancreas, aneurysms, etc.) [1,2]. In this case, it was ultimately the MRI that confirmed the diagnosis.

The purpose of the treatment is to interrupt the pathological cascade composed of weight loss, loss of retroperitoneal fat, narrowing of the aortomesenteric angle, external duodenal compression, vomiting, and weight loss. Therefore, its main goal is to promote progressive weight gain.

Most authors believe that conservative, medical treatment should be offered to patients with a short period of onset history, moderate symptoms, and incomplete duodenal obstruction [1]. Such measures include nasogastric tube insertion for gastric decompression, electrolyte and fluid correction, and nutritional management either in the form of a high-calorie oral diet if tolerated or postpyloric tube feeding. This nutritional support can be complemented by postural maneuvers and gut motility agents [9].

Surgical intervention is indicated in cases of conservative treatment failure, a prolonged disease with progressive weight loss, and recurrent upper gastrointestinal symptoms (at least once per week for more than six months). Duodenal distension and stasis, preference of the patient for surgical correction, and presence of complicated diseases such as peptic ulcers and pancreatitis due to biliary reflux are also reasons to opt for a surgical approach [1,7-8]. No time limit has yet been set to determine the medical treatment's ineffectiveness [9]. Nonetheless, some series have reported failure rates of 50%-70% [1]. In this case, the authors believed that considering the patient's severe symptoms, already causing renal disfunction, there would be no satisfactory response to conservative treatment on time. This was why, after discussion and consideration for the patient's expectations, the surgical treatment was decided to be the best fit. The operative options include Strong's procedure, gastrojejunostomy, and duodenojejunostomy [4]. 

Strong's procedure is preferably used in the pediatric population and consists of lysing the ligament of Treitz allowing for mobilization of the duodenum. Although being a simpler and less invasive procedure than the alternatives (no anastomoses are required), it has a high failure rate of 25% [9].

Gastrojejunostomy allows for gastric decompression, functioning as a bypass procedure, although not solving the duodenal obstruction [2]. This option has been progressively abandoned due to the increased incidence of its postoperative complications: blind loop syndrome, peptic stomal ulceration by biliary reflux, and recurrence of symptoms due to non-decompression of the duodenum [1,9].

Duodenojejunostomy was first introduced by Starley in 1910, and over the years, it has become the most frequent treatment with success rates of 90% [1]. The use of open and laparoscopic approaches has previously been reported with the latter presenting benefits like shorter hospitalization and less postoperative pain [4]. The first laparoscopic duodenojejunostomy was described in 1998 by Gersin and Heniford who proved this to be a safe, reproducible technique [8]. As Escaño et al. specified in their case series analysis, laparoscopic duodenojejunostomy is an effective minimally invasive treatment, with an acceptable rate of postoperative complications and favorable long-term results [5]. This is why it is considered the treatment of choice. In this case, the procedure was performed uneventfully and provided immediate symptomatic relief.

## Conclusions

Due to its rarity and nonspecific symptoms, Wilkie's syndrome poses a really challenging diagnosis. High index suspicion in cases of severe weight loss and upper gastrointestinal symptoms is of utmost importance. Enhanced CT is the gold standard diagnostic modality and should be employed whenever the patient presents with suggestive symptoms. Early detection can not only avoid the syndrome-associated complications but also improve the prognosis, making conservative measures more likely to be effective. Surgery should be considered in more severe, chronic cases or whenever medical treatment fails. Laparoscopic duodenojejunostomy has proven to be the best modality of choice warranting the best outcomes with a good safety profile.
